# Effects of mechanical ventilation with expiratory negative airway pressure on porcine pulmonary and systemic circulation: mechano-physiology and potential application

**DOI:** 10.1186/s12576-021-00801-5

**Published:** 2021-06-02

**Authors:** Mihoko Hagiwara-Nagasawa, Ryuichi Kambayashi, Ai Goto, Koki Chiba, Takeshi Wada, Yoshio Nunoi, Hiroko Izumi-Nakaseko, Yoshinori Takei, Akio Matsumoto, Keith G. Lurie, Atsushi Sugiyama

**Affiliations:** 1 Department of Pharmacology, Faculty of Medicine, Toho University, 5-21-16 Omori-nishi, Ota-ku, Tokyo, 143-8540 Japan; 2 Department of Cardiology, Juntendo University Urayasu Hospital, 2-1-1 Tomioka, Urayasu, Chiba 279-0021 Japan; 3 Department of Translational Research & Cellular Therapeutics, Faculty of Medicine, Toho University, 5-21-16 Omori-nishi, Ota-ku, Tokyo, 143-8540 Japan; 4 Department of Aging Pharmacology, Faculty of Medicine, Toho University, 5-21-16 Omori-nishi, Ota-ku, Tokyo, 143-8540 Japan; 5 Department of Emergency Medicine, University of Minnesota Medical School, Minneapolis, MN 55455 USA

**Keywords:** Cardiovascular collapse, Expiratory negative airway pressure, Hemodynamics, Mechano-physiology, Resuscitation

## Abstract

We studied the impact of mechanically regulated, expiratory negative airway pressure (ENAP) ventilation on pulmonary and systemic circulation including its mechanisms and potential applications. *Microminipigs* weighing about 10 kg were anesthetized (*n* = 5). First, hemodynamic variables were evaluated without and with ENAP to approximately −16 cmH_2_O. ENAP significantly increased heart rate and cardiac output, but decreased right atrial, pulmonary arterial and pulmonary capillary wedge pressures. Second, the evaluation was repeated following pharmacological adrenergic blockade, modestly blunting ENAP effects. Third, fluvoxamine (10 mg/kg) was intravenously administered to intentionally induce cardiovascular collapse in the presence of adrenergic blockade. ENAP was started when systolic pressure was < 40 mmHg in the animals assigned to ENAP treatment-group. Fluvoxamine induced cardiovascular collapse within 4 out of 5 animals. ENAP increased systolic pressure to > 50 mmHg (*n* = 2): both animals fully recovered without neurological deficit, whereas without ENAP both animals died of cardiac arrest (*n* = 2). ENAP may become an innovative treatment for drug-induced cardiovascular collapse.

## Background

A severe low cardiac-output state requires assist devices including intra-aortic balloon pump, ventricular assist device and extracorporeal circulation to avoid life-threatening end-organ hypoperfusion [1]. Although these devices can be effective for ameliorating the cardiovascular collapse, their application may be time-consuming, highly invasive and expensive with less versatility. The positive airway pressure as an alternative mechanical approach has been applied to recruit lung tissue, thus improving oxygenation in the severe acute respiratory and heart failure that includes the positive end-expiratory pressure, continuous positive airway pressure and adaptive servo-ventilation [2–4]. However, these positive airway-pressure therapies are known to attenuate the intrathoracic vacuum associated with normal breathing and reduce venous return and cardiac output [5–7]. Adaptive servo-ventilation was found to potentially worsen the prognosis of patients having central sleep apnea with systolic heart failure [8].

Over a past quarter century, a greater understanding of heart–brain–lung interactions has resulted in novel resuscitation methods and technologies, significantly improving outcomes from cardiac arrest, which include recent approaches to regulate intrathoracic pressure [9]. Indeed, “inspiratory” negative airway pressure with impedance threshold device has been shown to increase the cardiac output during cardiac arrest resuscitation with cardiac massage [10–13]. More recently, another technology with an intrathoracic pressure regulator was developed, which actively creates a continuous low level of negative intrathoracic pressure during cardiac massage following each inspiratory positive pressure breath [14–16]. Accordingly, short term use of “expiratory” negative airway pressure (ENAP) ventilation has been shown to improve spontaneous systemic circulation during clinical as well as experimental non-cardiac arrest shock like hemorrhagic hypovolemic states [17–19]. Since the benefits of ENAP are not completely understood, we primarily focused on mechano-physiology of this new treatment; namely, how ENAP may affect pulmonary as well as systemic circulation, whether the effects of ENAP may depend on adrenergic activity, and whether ENAP can be effective against the drug-induced cardiovascular collapse as a non-invasive treatment.

**Fig. 1:**
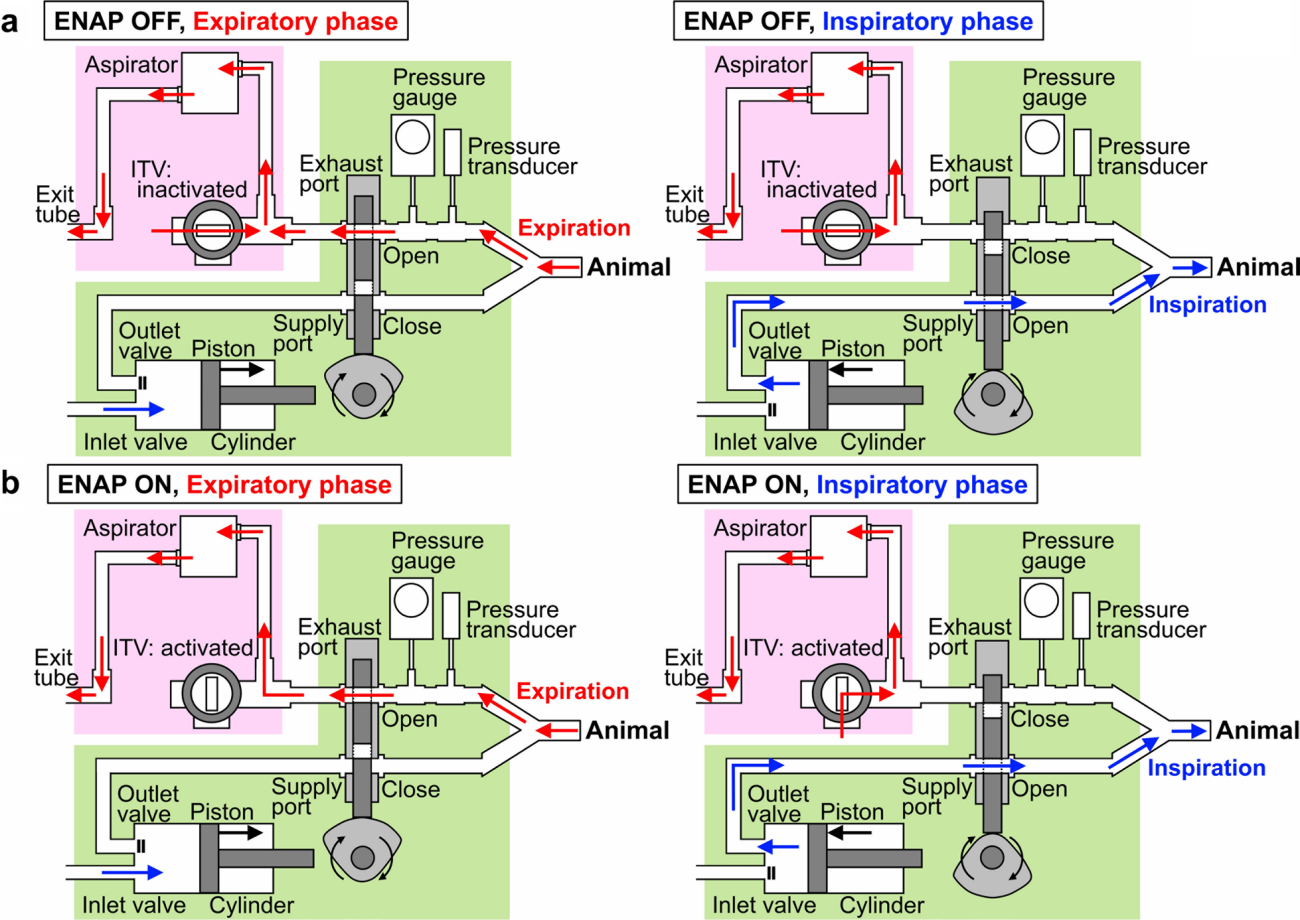
Schematic representation of standard ventilation circuit along with the generation system of expiratory negative airway pressure (ENAP). Green and pink panels indicate standard ventilation circuit and generation system of ENAP, respectively. **a** Airflows (red and blue arrows) are shown during the expiratory (left) and inspiratory (right) phases for the animal when the impedance threshold valve (ITV) is inactivated (ENAP OFF). **b** Airflows (red and blue arrows) are shown during the expiratory (left) and inspiratory (right) phases when the ITV is activated (ENAP ON). Black arrows show the directions of moves of piston and fan-shaped pulley

In this study, we initially developed a novel ENAP generation system which can be used with the standard mechanical ventilator as well as the manual ventilator such as a bag valve mask device (Fig. 1). Secondly, the effects of ENAP on the pulmonary and systemic circulation were assessed under the physiologically controlled condition using anesthetized *microminipigs*. Thirdly, the assessment was repeated after pharmacological adrenoceptor blockade to assess how the sympathetic nervous system may alter the effects of ENAP treatment. Lastly, we performed a pilot translational medicine study to determine if ENAP can be used to treat cardiovascular collapse from a drug overdose with antidepressant fluvoxamine. In our previous study using the halothane-anesthetized beagle dogs [20], intravenous administration of fluvoxamine in a dose of 10 mg/kg significantly decreased the ventricular contraction as well as the total vascular resistance; resulting in a severe hypotension possibly through the Ca^2+^ and Na^+^ channels inhibition. Since fluvoxamine is one of the antidepressant drugs that can be used for suicide to commit [21, 22], its toxic dose of 10 mg/kg was administered in the setting of α- and β-adrenoceptor blockade to mimic clinically ill situations where ENAP ventilation might be applied.

## Methods

### Animals

Experiments were performed in 5 male *microminipigs* (Fuji Micra Inc., Shizuoka, Japan) weighing approximately 10 kg, which are small-sized miniature pigs developed for laboratory use [23]. The animals were individually housed in stainless steel cages on a 12 h light (6:00–18:00)–dark (18:00–6:00) cycle, and were given 200 g/day of standard pellet diet (MMP pellets, Marubeni Nisshin Feed Co. Ltd., Tokyo, Japan) and free access to tap water. The animal rooms were maintained at a temperature of 23 ± 2 °C and a relative humidity of 50 ± 20%.

### Induction of general anesthesia

*Microminipigs* were pre-anesthetized with an intramuscular injection of ketamine (16 mg/kg) and xylazine (1.6 mg/kg). A 24G cannula was introduced into a superficial auricular vein followed by an anesthetic injection of 1 mg/kg of propofol. Then, the animal was positioned supine. After intubation with a 6-mm cuffed endotracheal tube, anesthesia was maintained by halothane inhalation (1% v/v) vaporized in oxygen with a volume-limited ventilator (SN-480-3; Shinano Manufacturing Co., Ltd., Tokyo, Japan) [24–26]. Tidal volume and respiratory rate were set at 10 mL/kg and 15 breaths/min, respectively. Oxygen saturation was measured using a pulse oximeter (OLV-2700; Nihon Kohden Corporation, Tokyo, Japan) with disposable probe.

### Surgical preparations

Two 6F-size catheter sheaths (FAST-CATHTM 406104; St. Jude Medical, Daig Division, Inc., Minnetonka, MN, USA) were used; one was inserted into the right femoral artery toward aorta, and the other was done into the right femoral vein toward inferior vena cava. Heparin calcium (100 IU/kg) was administered to prevent the blood clotting through a flush line of the catheter sheath placed at the right femoral vein. A 5F-size catheter was inserted into the aorta through the catheter sheath at the right femoral artery, tip of which was positioned at a level of diaphragm to measure the aortic pressure. A triple lumen thermodilution balloon catheter (132F5; Edwards Lifesciences, Irvine, CA, USA) was inserted into the right side of the heart through the catheter sheath placed at the right femoral vein to measure the right atrial pressure (RAP), pulmonary arterial systolic pressure (PAP), pulmonary capillary wedge pressure (PCWP) and cardiac output. The coronary arterial perfusion pressure (CPP) was calculated by subtracting the RAP from the diastolic aortic pressure. The systemic perfusion pressure and pulmonary perfusion pressure were calculated by subtracting the RAP from the mean blood pressure and the PCWP from the mean PAP, respectively. The cardiac output was measured by using a standard thermodilution method with a cardiac output computer (MFC-1100; Nihon Kohden Corporation). The systemic vascular resistance (SVR) and pulmonary vascular resistance (PVR) were calculated by dividing the systemic perfusion pressure and pulmonary perfusion pressure by the cardiac output, respectively. The electrocardiogram was obtained from the A–B lead. The electrocardiogram and cardiohemodynamic variables were continuously monitored with a polygraph system (RM-6000; Nihon Kohden Corporation).

### Preparation of ENAP circuit

Figure 1 indicates schema of standard ventilation circuit (green panels) along with the generation system of ENAP (pink panels). During the expiratory phase for the animal (Fig. 1a left), the expiration gas was guided by the opened exhaust port (red arrows) with the supply port closed, whereas the anesthetic gas was drawn into the cylinder (blue arrows) by the piston through the inlet valve with the outlet valve closed in the standard ventilation circuit (green panel). On the other hand, during the inspiratory phase for the animal (Fig. 1a right), the anesthetic gas was pushed out by the piston from the cylinder (blue arrows) into the animal through the opened outlet valve and supply port with the exhaust port and inlet valve closed (green panel).

The generation system of ENAP consists of a custom-made, one-way impedance threshold valve (ITV), an aspirator as a vacuum source (DAS-01, AS ONE Corporation, Osaka, Japan) and connecting tubes, which were placed between the exhaust port and the exit tube (Fig. 1, pink panels). An on/off stopcock valve was equipped in the ITV, which can be used to bypass the channel occluded by the valve. When the ITV was inactivated (ENAP OFF), during the expiratory phase (Fig. 1a left) tidal volume of expiratory gas from the animal was drawn to the exit tube through the aspirator with no expiratory resistance. The pressure within the connecting tube was kept at the atmospheric air pressure during both expiratory and inspiratory phases, since the ITV can completely bypass the airflow without any resistance. The intra-tracheal airway pressure was estimated by using the pressure transducer along with the pressure gauge, which were placed between the endotracheal tube and the exhaust port. The atmospheric air pressure was set 0 cmH_2_O of intra-tracheal airway pressure.

When the ITV was activated (ENAP ON) (Fig. 1b right, pink panel), the negative pressure gradually increased within the expiratory airway tube separated by the exhaust port, aspirator and ITV toward − 25 cmH_2_O by the aspirator, since the ITV did not bypass the airflow. When the negative airway pressure exceeded − 25 cmH_2_O (< − 25 cmH_2_O), the threshold valve in the ITV automatically opened, which kept the pressure in the expiratory airway tube below or equal to − 25 cmH_2_O (≥ − 25 cmH_2_O) without changing the function of the exhaust port, aspirator and ITV (Fig. 1b right, pink panel) [27]. During the expiratory phase (Fig. 1b left), the tidal volume of expiratory gas from the animal was drawn to the exit tube by the aspirator. The pressure within the connecting tube became negative during expiratory phase, since the ITV functioned to impede the airflow. During the next inspiratory phase, the exhaust port was closed, so that the generator system can prepare the negative pressure of − 25 cmH_2_O (Fig. 1b right, pink panel). Meanwhile, the gas was pushed out by the piston from the cylinder through the supply port to the animal, cancelling the negative airway pressure which had been developed during the preceding expiratory phase.

### Experimental protocol

**Fig. 2:**
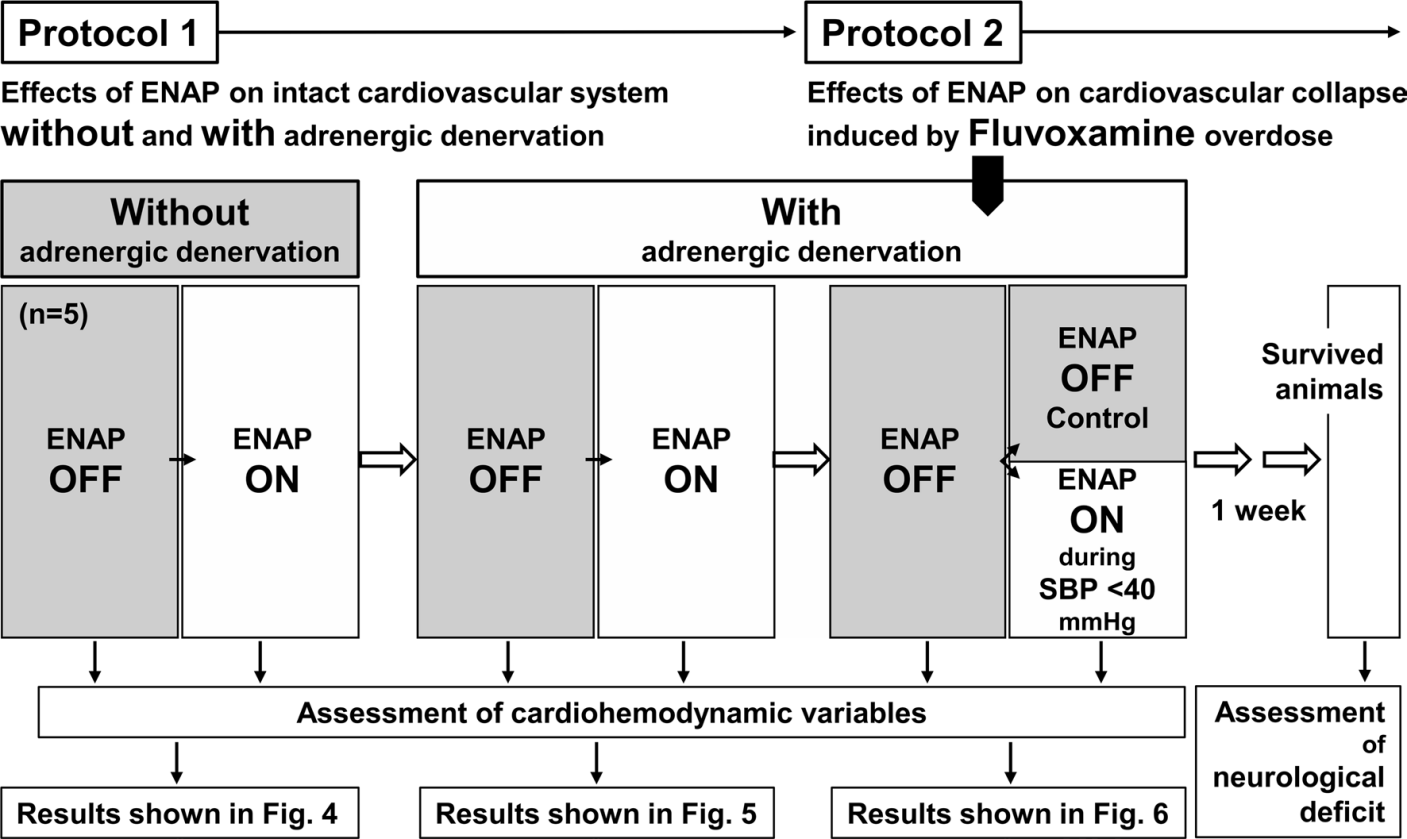
Schematic representation of protocol characteristics, in which each procedure was performed in series using 5 animals. ENAP: expiratory negative airway pressure; and SBP: systolic blood pressure. Adrenergic denervation was initially performed by phentolamine (1 mg/kg, i.v.) and propranolol (0.3 mg/kg, i.v.)

Schematic representation of protocol characteristics is shown in Fig. 2.

#### Protocol 1

Effects of ENAP on the cardiovascular system of intact animals were assessed without and with adrenergic denervation. The intra-tracheal airway pressure was obtained at the end of the inspiratory and expiratory phases in successive three respiratory cycles, whereas the heart rate, aortic pressure, RAP, PAP, PCWP, CPP, systemic perfusion pressure, pulmonary perfusion pressure, SVR and PVR were measured using the last beat at the end of each respiratory phase in a similar manner to those of intra-tracheal airway pressure. The cardiac output was measured 3 times irrespective of the respiratory phases, since it took about 10 s to calculate the cardiac output, which was > 2 times of a respiratory cycle of 4 s. After confirming the stability of the hemodynamic condition, the airway pressure and each of the cardiovascular variables were recorded without ENAP during the inspiratory and expiratory phases. Next, ENAP was added, and after 1 min to allow for the full effect, each measurement was repeated. Finally, 1 mg/kg of phentolamine and 0.3 mg/kg of propranolol were intravenously administered to pharmacologically block the sympathetic nerve. After 1 min to allow for the full pharmacological effect, noradrenaline (0.3 μg/kg) and isoproterenol (0.03 μg/kg) was intravenously administered to confirm α- and β-adrenoceptor blockade, respectively. After confirming that the maximum elevations of the mean blood pressure and heart rate by noradrenaline and isoproterenol were < 10 mmHg and < 10 bpm, respectively, each variable was recorded without and with ENAP in the same manner as those before the pharmacological treatment.

#### Protocol 2

Effects of ENAP on the cardiovascular collapse induced by fluvoxamine overdose were assessed. Approximately 30 min after finishing the protocol 1, a dose of 0.3 μg/kg of noradrenaline was intravenously administered to confirm α-adrenoceptor blocking action of phentolamine, whereas a dose of 0.03 μg/kg of isoproterenol was done to verify β-adrenoceptor blocking action of propranolol. When noradrenaline elevated the mean blood pressure by > 10 mmHg, additional 1 mg/kg of phentolamine was intravenously administered. Also, when isoproterenol increased the heart rate by > 10 bpm, 0.3 mg/kg of propranolol was intravenously administered. Then, the basal systolic blood pressure and heart rate were measured, whereas electrocardiogram was continuously monitored. Next, fluvoxamine maleate in a dose of 10 mg/kg was intravenously infused over 10 min, and the systolic blood pressure and heart rate were assessed every 1 min after the start of administration (*n* = 5). The animals were randomly assigned to 2 groups; namely, control group and ENAP-treated group. When the systolic blood pressure decreased to < 40 mmHg, the ENAP was started for the animals assigned to the ENAP-treated group, which was terminated when the systolic blood pressure increased to > 50 mmHg as a general rule, whereas the ENAP treatment was not applied for the other animals assigned to the control group.

### Drugs

Fluvoxamine maleate was extracted from a commercially available tablet (Luvox®, AbbVie GK, Tokyo, Japan) with distilled water in a concentration of 10 mg/mL. Phentolamine (Regitin® inj, Novartis Pharma K.K., Tokyo, Japan) was diluted with saline to concentration of 3.33 mg/mL. Propranolol (Inderal® inj, AstraZeneca K.K., Osaka, Japan), isoproterenol (Proternol-L inj, Kowa Company, Ltd., Nagoya, Japan), noradrenaline (Nor-adrenalin inj, Daiichi Sankyo Co., Ltd., Tokyo, Japan), ketamine (Ketalar®, Daiichi Sankyo Co., Ltd.), xylazine (Celactal®, Bayer Healthcare Co., Tokyo, Japan), propofol (propofol inj [FK], Frensenius Kabi Japan K.K., Tokyo, Japan), halothane (Fluothane®, Takeda Pharmaceutical Co., Ltd., Osaka, Japan) and heparin calcium (Caprocin®, Sawai Pharmaceutical Co., Ltd., Osaka, Japan) were purchased.

### Statistical analysis

Data are presented as mean ± standard error of means (SEM). The statistical significances were evaluated with paired *t*-test for cardiac output between ENAP OFF and ON, and for each parameter at ENAP OFF between without and with adrenergic denervation. Those within a parameter except for the cardiac output were evaluated by two-way, repeated measures analysis of variance (ANOVA) followed by Bonferroni multiple comparison. A *p*-value < 0.05 was considered to be statistically significant.

## Results

### Protocol 1

Oxygen saturation remained ≥ 99% with supplemental O_2_ in each animal, whereas no animal exerted hemodynamic collapse leading to its death during protocol 1.

#### Effects of ENAP on pulmonary and systemic circulation in animals without pharmacological adrenergic denervation

**Fig. 3:**
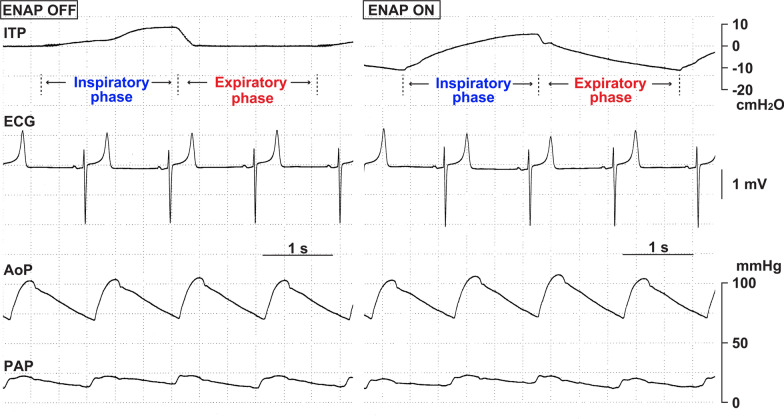
Typical tracings of the intra-tracheal pressure (ITP), surface lead A–B electrocardiogram (ECG), aortic blood pressure (AoP) and pulmonary arterial pressure (PAP) without expiratory negative airway pressure (ENAP) (ENAP OFF, left) and with ENAP (ENAP ON, right)

**Fig. 4:**
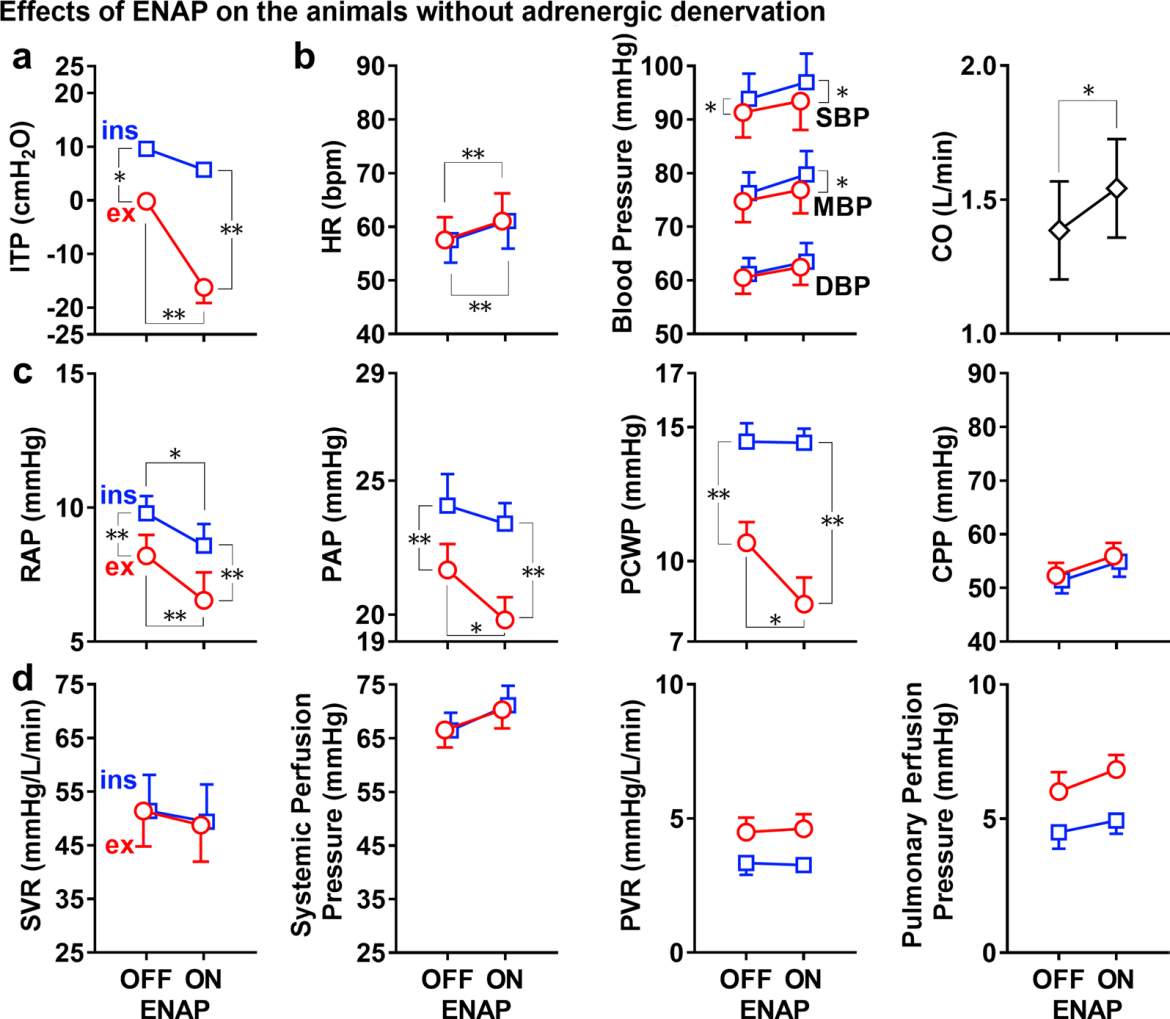
Summary of the effects of expiratory negative airway pressure (ENAP) on the intra-tracheal pressure (ITP) (**a**, top left panel); the heart rate (HR), systolic/mean/diastolic aortic blood pressures (SBP/MBP/DBP) and cardiac output (CO) (**b**, top right three panels); the right atrial pressure (RAP), pulmonary arterial systolic pressure (PAP), pulmonary capillary wedge pressure (PCWP) and coronary artery perfusion pressure (CPP) (**c**, middle four panels); and the systemic vascular resistance (SVR), systemic perfusion pressure, pulmonary vascular resistance (PVR) and pulmonary perfusion pressure (**d**, bottom four panels) in the animals without pharmacological adrenergic denervation. Squares: end-inspiratory (ins); circles: end-expiratory (ex); OFF: without ENAP; and ON: with ENAP. Data are presented as mean ± SEM (*n* = 5). *p < 0.05 and **p < 0.01

Typical tracings of intra-tracheal pressure, electrocardiogram, aortic pressure and pulmonary arterial pressure without and with ENAP are depicted in Fig. 3 left and right panels, respectively. Effects of ENAP on the intra-tracheal pressure are summarized in Fig. 4a. Without ENAP, the intra-tracheal pressures at the end-inspiratory and end-expiratory phases were 9.6 ± 0.7 and − 0.2 ± 0.2 cmH_2_O, respectively. The intra-tracheal pressure was significantly lower at the end-expiratory phase than that at the end-inspiratory phase. ENAP tended to lower the intra-tracheal pressures at end-inspiratory phase to 5.7 ± 0.5 cmH_2_O and significantly lowered the pressure at end-expiratory phase to − 16.2 ± 2.9 cmH_2_O, of which decrement was greater in the latter (− 16.0 cmH_2_O) than in the former (− 3.9 cmH_2_O).

Effects of ENAP on the heart rate, blood pressure and cardiac output are summarized in Fig. 4b. Without ENAP, the heart rate and systolic/mean/diastolic blood pressures were 57 ± 4 bpm and 93.8 ± 4.7/76.3 ± 3.9/61.2 ± 3.0 mmHg at the end-inspiratory phase, whereas those were 58 ± 4 bpm and 91.3 ± 4.7/74.7 ± 3.9/60.5 ± 3.0 mmHg at the end-expiratory phase, respectively. The systolic blood pressure was significantly lower at the end-expiratory phase than that at the end-inspiratory phase. The cardiac output was 1.39 ± 0.18 L/min, which is within normal range of the cardiac output for intact *microminipigs*. ENAP significantly increased the heart rate by 4 bpm at both phases and the cardiac output by 0.16 L/min, and tended to increase each of the systolic/mean/diastolic blood pressures.

Effects of ENAP on the RAP, PAP, PCWP and CPP are summarized in Fig. 4c. Without ENAP, they were 9.8 ± 0.6, 24.1 ± 1.2, 14.5 ± 0.7 and 51.4 ± 2.4 mmHg at the end-inspiratory phase and 8.2 ± 0.8, 21.7 ± 1.0, 10.7 ± 0.8 and 52.3 ± 2.4 mmHg at the end-expiratory phase, respectively. The RAP, PAP and PCWP were significantly lower at the end-expiratory phase than those at the end-inspiratory phase, whereas no significant difference was observed in the CPP between the phases. ENAP decreased the RAP at both phases by 1.2 and 1.7 mmHg, respectively. Also, it reduced the PAP and PCWP at the end-expiratory phase by 1.9 and 2.3 mmHg, respectively. Meanwhile, it tended to increase the CPP at both phases by 3.5 and 3.6 mmHg, respectively.

Effects of ENAP on the SVR, systemic perfusion pressure, PVR and pulmonary perfusion pressure are summarized in Fig. 4d. Without ENAP, they were 51.4 ± 6.7 mmHg/L/min, 66.5 ± 3.3 mmHg, 3.3 ± 0.4 mmHg/L/min and 4.5 ± 0.6 mmHg at the end-inspiratory phase and 51.4 ± 6.6 mmHg/L/min, 66.5 ± 3.3 mmHg, 4.5 ± 0.5 mmHg/L/min and 6.0 ± 0.7 mmHg at the end-expiratory phase, respectively. The PVR and pulmonary perfusion pressure tended to be lower at the end-inspiratory phase than those at the end-expiratory phase. ENAP tended to increase the systemic perfusion pressure at the both phases. It also tended to increase the pulmonary perfusion pressure but tended to decrease the SVR at both phases, whereas it did not alter the PVR at either phase.

#### Effects of ENAP on pulmonary and systemic circulation in animals with pharmacological adrenergic denervation

**Fig. 5:**
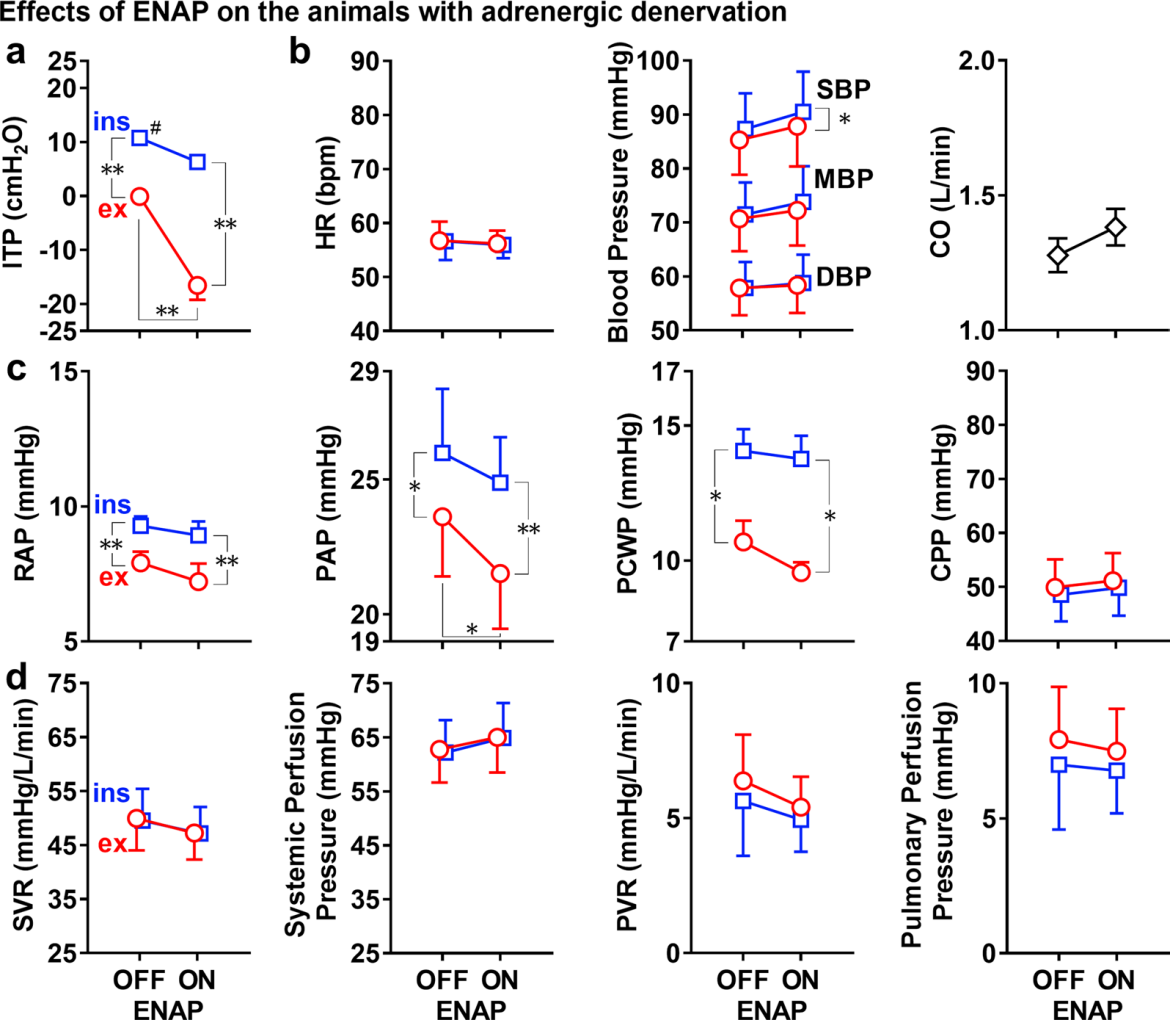
Summary of the effects of expiratory negative airway pressure (ENAP) on the intra-tracheal pressure (ITP) (**a**, top left panel); the heart rate (HR), systolic/mean/diastolic aortic blood pressures (SBP/MBP/DBP) and cardiac output (CO) (**b**, top right three panels); the right atrial pressure (RAP), pulmonary arterial systolic pressure (PAP), pulmonary capillary wedge pressure (PCWP) and coronary artery perfusion pressure (CPP) (**c**, middle four panels); and the systemic vascular resistance (SVR), systemic perfusion pressure, pulmonary vascular resistance (PVR) and pulmonary perfusion pressure (**d**, bottom four panels) in the animals with pharmacological adrenergic denervation by phentolamine (1 mg/kg, i.v.) and propranolol (0.3 mg/kg, i.v.). Squares: end-inspiratory (ins); circles: end-expiratory (ex); OFF: without ENAP; and ON: with ENAP. Data are presented as mean ± SEM (*n* = 5). *p < 0.05 and **p < 0.01. ^#^*p* < 0.05 vs. before adrenergic denervation

Effects of ENAP on the intra-tracheal pressure are summarized in Fig. 5a. Without ENAP, the intra-tracheal pressures at the end-inspiratory and end-expiratory phases were 10.8 ± 1.0 and − 0.1 ± 0.4 cmH_2_O, respectively. The adrenergic denervation increased the former by 1.2 cmH_2_O (from 9.6 to 10.8 cmH_2_O), but it hardly altered the latter (from − 0.2 to − 0.1 cmH_2_O). The intra-tracheal pressure was significantly lower at the end-expiratory phase than that at the end-inspiratory phase. ENAP tended to lower the intra-tracheal pressures at end-inspiratory phase to 6.3 ± 0.6 cmH_2_O and significantly lowered it at end-expiratory phase to − 16.5 ± 2.7 cmH_2_O, of which decrement was greater in the latter (− 16.4 cmH_2_O) than in the former (− 4.5 cmH_2_O).

The effects of ENAP on the heart rate, blood pressure and cardiac output are summarized in Fig. 5b. Without ENAP, the heart rate and systolic/mean/diastolic blood pressures were 57 ± 3 bpm and 87.3 ± 6.6/71.5 ± 5.9/57.8 ± 4.9 mmHg at the end-inspiratory phase, whereas those were 57 ± 4 bpm and 85.3 ± 6.5/70.7 ± 6.0/57.8 ± 5.0 mmHg at the end-expiratory phase, respectively. No significant difference was observed in the blood pressures between the phases. The cardiac output was 1.28 ± 0.06 L/min. The effects of ENAP on the blood pressure and cardiac output were similar to those before the adrenergic blockade but blunted, whereas the ENAP-induced positive chronotropic effect disappeared.

Effects of ENAP on the RAP, PAP, PCWP and CPP are summarized in Fig. 5c. Without ENAP, they were 9.3 ± 0.3, 26.0 ± 2.4, 14.1 ± 0.8 and 48.6 ± 5.0 mmHg at the end-inspiratory phase and 7.9 ± 0.4, 23.6 ± 2.2, 10.7 ± 0.8 and 49.9 ± 5.2 mmHg at the end-expiratory phase, respectively. The PAP was tended to be increased by the adrenergic denervation by 1.9 mmHg at both phases. The RAP, PAP and PCWP were significantly lower at the end-expiratory phase than those at the end-inspiratory phase, whereas no significant difference was observed in the CPP between the phases. The effects of ENAP on the RAP, PAP, PCWP and CPP were similar to those before the adrenergic blockade but blunted except that those on PAP were enhanced.

Effects of ENAP on the SVR, systemic perfusion pressure, PVR and pulmonary perfusion pressure are summarized in Fig. 5d. Without ENAP, they were 49.5 ± 5.9 mmHg/L/min, 62.2 ± 6.0 mmHg, 5.6 ± 2.0 mmHg/L/min and 7.0 ± 2.4 mmHg at the end-inspiratory phase and 50.0 ± 5.9 mmHg/L/min, 62.8 ± 6.1 mmHg, 6.4 ± 1.7 mmHg/L/min and 7.9 ± 2.0 mmHg at the end-expiratory phase, respectively. The adrenergic denervation tended to increase the PVR and pulmonary perfusion pressure, and its reverse was true for the systemic perfusion pressure, whereas it little impacted the SVR.

An interaction effect was observed between respiratory phase (ins/ex) and ENAP (ON/OFF) in the intra-tracheal pressure (*p* = 0.0058), PAP (0.0308) and PCWP (0.0148) in the absence of adrenergic denervation, which was not detected in the other variables. On the other hands, after adrenergic denervation an interaction effect was detected only in the intra-tracheal pressure (*p* = 0.0012). When the main effects [respiratory phase (ins/ex) and ENAP (ON/OFF)] were statistically significant, each result obtained by the post-hoc test was qualified with taking their interactions into account.

### Protocol 2

#### Effects of ENAP on the fluvoxamine-induced cardiovascular collapse

**Fig. 6:**
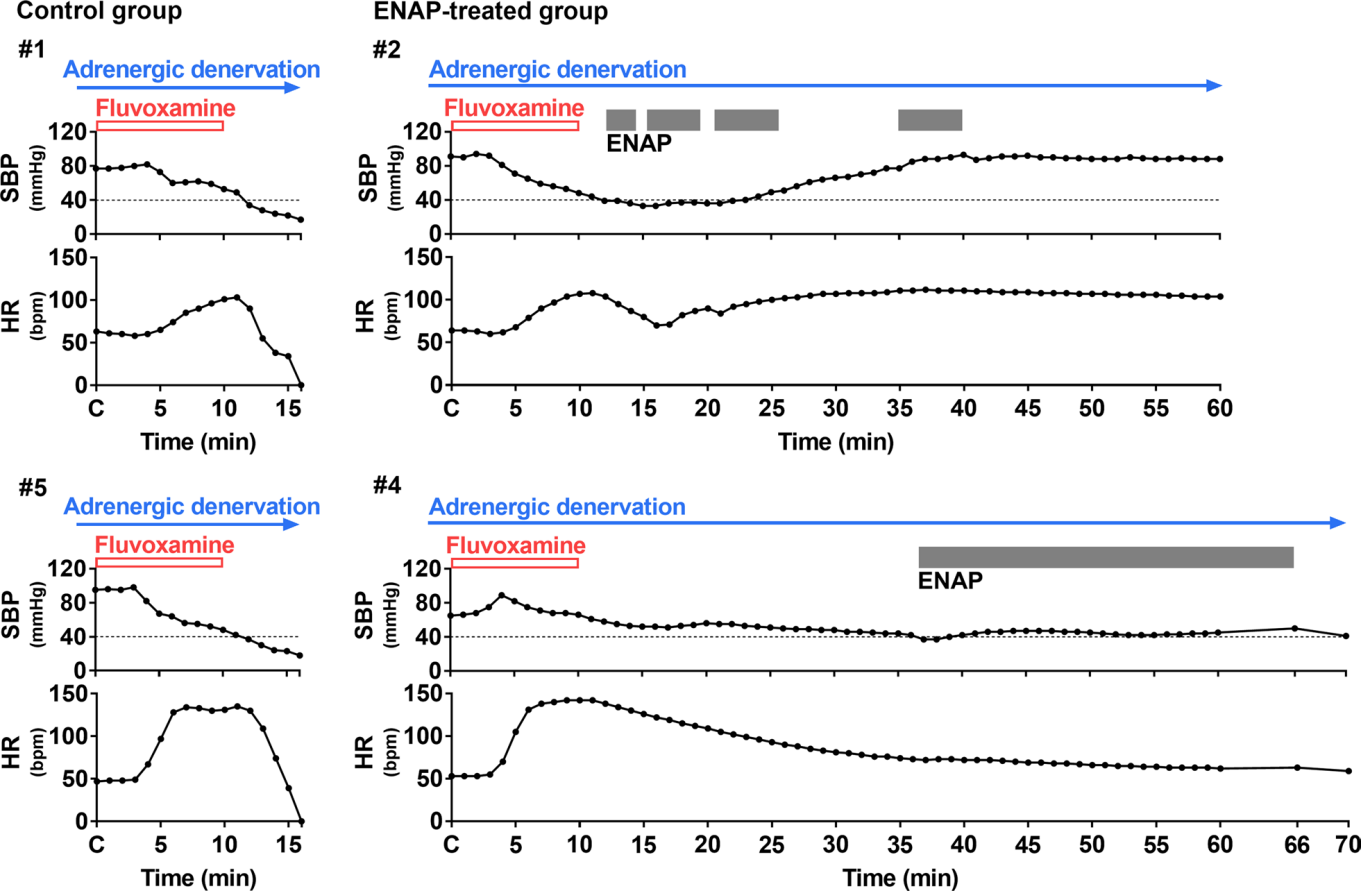
The effects of expiratory negative airway pressure (ENAP) on the systolic aortic blood pressure (SBP) and heart rate (HR) during fluvoxamine-induced cardiovascular collapse in each animal. In the presence of pharmacological adrenergic denervation, 10 mg/kg of fluvoxamine maleate decreased the SBP to < 40 mmHg within 4 animals (animals #1, #2, #4 and #5), whereas the SBP was kept > 40 mmHg in 1 animal (#3; not shown in the figure) during the observation period. When the SBP decreased to < 40 mmHg, the ENAP treatment was applied to animals #2 and #4 (ENAP-treated group, *n* = 2, right panels), whereas it was not used for animals #1 and #5 (Control group, *n* = 2, left panels). Gray bar indicates the period when the animals received the ENAP treatment. Note that the SBP was effectively elevated by the ENAP treatment during cardiovascular collapse (#2, #4) as well as at the recovery phase (#2); thus, the ENAP saved the lives of the animals in fatal cardiovascular collapse. C: Control value before the administration of fluvoxamine maleate

One mg/kg of phentolamine was supplemented to 3 animals (#1, #3 and #4), whereas 0.3 mg/kg of propranolol was done to 2 ones (#1 and #4). The basal systolic aortic pressure and heart rate (C) were 85 ± 6 mmHg and 55 ± 3 bpm before the administration of fluvoxamine, respectively (*n* = 5). Fluvoxamine decreased the systolic aortic pressure to < 40 mmHg along with the reduction of O_2_ saturation to < 80% within 4/5 animals (#1, #2, #4 and #5). The time courses of effects of ENAP on the systolic aortic pressure and heart rate in each animal during the fluvoxamine-induced cardiovascular collapse are summarized in Fig. 6. In the control group (*n* = 2; #1 and #5), the systolic blood pressure rapidly decreased to < 40 mmHg after the start of drug administration, resulting in irreversible cardiohemodynamic collapse followed by the cardiac arrest by 16 min. Meanwhile in the ENAP-treated group (*n* = 2; #2 and #4), ENAP effectively increased the systolic aortic pressure to > 50 mmHg along with the elevation of O_2_ saturation to > 90%, which survived after the experimental protocol. No neurological deficit was observed in either of these animals (#2 and #4) at 1 week after the experiment.

## Discussion

In order to study mechanisms of ENAP treatment and its potential clinical benefit for supporting systemic circulation during cardiovascular collapse, we assessed the hemodynamic effects of this new treatment modality without and with pharmacological adrenoceptor blockade, and then performed a pilot survival study to obtain the proof of concept of its therapeutic strategy. We could not make a scientific judgment on the utility of ENAP ventilation against cardiovascular collapse solely based on a small number of samples in protocol 2. However, we believe that pharmacological analysis of the effects of ENAP ventilation on the pulmonary and systemic circulation in protocol 1 along with a pilot translational study against cardiovascular collapse in protocol 2 would at least in part provide a basic knowledge to further investigate its clinical utility as discussed below.

### Effects of ENAP on pulmonary and systemic circulation in intact *microminipigs*

Before the adrenergic blockade, ENAP reduced the RAP, PAP and PCWP by augmentation of the intrathoracic vacuum, enhancing the venous return as well as the orthodromic forward flows, which could explain increase of the cardiac output. These observations on ENAP could be expected by physiological and/or pathophysiological knowledge; however, they have not been reported before and may provide rationale for explaining its mechanism. Although ENAP-induced changes in the intra-tracheal pressure were essentially the same between without and with the adrenergic blockade, ENAP-induced cardiovascular responses like the increases of the heart rate and cardiac output as well as the decreases of the RAP and PCWP tended to be blunted after the adrenergic blockade. For example, ENAP-dependent increase of the cardiac output was 0.16 L/min before the adrenergic blockade, which decreased to 0.11 L/min after the blockade. Thus, the effects of ENAP on pulmonary and systemic circulation were dependent, at least in part, upon sympathetic activity.

The heart rate was increased by ENAP before adrenergic blockade, of which causal link deserves a comment. The following 2 mechanisms can be proposed; namely, one is that an increased venous return may stretch the atrium, enhancing the stretch-activated inward current in the sinus nodal area; and the other is that an atrial stretch receptor-mediated, nervous reflex would stimulate the sinus nodal automaticity, so called the *Bainbridge reflex* [28]. Since there was no increase in the heart rate with ENAP after the adrenergic blockade, sympathetic nervous system may play an important role in the ENAP-induced positive chronotropic effect. Moreover, one can speculate such an alternative pathway may be present that ENAP-induced negative intrathoracic pressure by itself might augment the cardiac sympathetic afferent reflex, which would in turn influence cardiovascular variables through sympathetic outflow tract.

The cardiac output is determined by the product of the heart rate and stroke volume, and ENAP by itself would be expected to directly increase the latter based on the physiological and/or pathophysiological theories as discussed above. Thus, it is no wonder that ENAP tended to increase the cardiac output even when the heart rate did not increase after adrenergic blockade. In addition, it is noteworthy that the adrenergic denervation increased the intra-tracheal pressure at the end-inspiratory phase from 9.6 to 10.8 cmH_2_O in the absence of ENAP (# in Fig. 5) along with the tendency to elevate the PAP at both phases, suggesting that the constitutively active adrenergic tone may benefit the respiratory function as well as pulmonary circulation.

### Effects of ENAP on the drug-induced cardiovascular collapse in *microminipigs*

Since ENAP ventilation must work in the setting of an overdose of various kinds of drugs simultaneously inducing α-and/or β-adrenoceptor blockade, we administered a toxic dose of fluvoxamine to *microminipigs* after the adrenergic blockade, which induced cardiovascular collapse within 4 animals out of 5. Without ENAP, 2 animals out of 2 developed cardiac arrest and died, whereas with ENAP, 2 animals out of 2 survived without any neurological deficit. One pig failed to develop a systolic blood pressure < 40 mmHg, so it was not randomized/assigned to the treatment. This pilot study analyzing the effects of ENAP on survival after the sympathetic denervation and drug overdose with fluvoxamine suggests that ENAP may increase the possibility for survival. The neurologically sound survival may indirectly support the previously described hypothesis that the negative intrathoracic pressure could be transduced through the paravertebral venous/epidural plexus and spinal fluid to the intracranial compartment, which could attenuate the increased intracranial pressure potentially occurring during cardiovascular collapse [29, 30]. The sample size was small, but good reproducibility of results shown in the current study indicates that ENAP can be effective against the drug-induced cardiovascular collapse as a non-invasive treatment.

## Limitations

First, pilot survival study in protocol 2 is suggestive but a larger sample size is needed for establishing a more definitive result; indeed, the current study may have been underpowered to examine the full impact of ENAP in the presence of sympathetic denervation. We have started follow-up experiments for the survival study as another project to make up such the limitation. Second, negative airway pressure can occur in patients during inspiratory phase of spontaneous breathing, which may potentially attenuate the magnitude of cardiovascular collapse. Thus, another series of project is now on-going to directly compare the survival rate between ENAP ventilation and spontaneously breathing condition using the cardiovascular collapse model before starting its clinical application. Third, it is unknown how rapidly or how much extent the vacuum should be applied: in other words, we do not yet know the ideal negative slope of the intra-tracheal pressure during expiratory phase although we applied slower onset of 2 s but greater vacuum of about –16 cmH_2_O to the animals than those reported before that were 1.2 s and − 12 cmH_2_O, respectively [17–19]. Fourth, it is also unknown what implications there are for the lungs injuries, although no obvious or subtle ones were confirmed in this study. Since O_2_ saturation remained ≥ 99% with supplemental O_2_ and no significant increase of the PVR was induced by ENAP in the intact animals during protocol 1, we did not look at this issue in detail. Others have checked the lungs injuries in the setting of hemorrhagic shock and there were no obvious or subtle lungs injuries in those studies either [17–19]. Fifth, ENAP ventilation may not be able to be used for patients with lung injury, since in a recent study using a porcine polytrauma model of hemorrhagic shock and acute lung injury, the use of intrathoracic pressure regulator inducing ENAP was shown to compromise pulmonary function without significantly improving hemodynamic variables [31]. Accordingly, there might be a room for clinical development of ENAP ventilation at least for patients who do not have lung injury but suffer from severe cardiovascular collapse. Sixth, it would not be recommended for patients with pathophysiology requiring treatment with the positive airway pressure, including chronic obstructive pulmonary disease, atelectasis and substantial hypoxemia.

## Conclusion

The current study helps to clarify some of mechano-physiology and potential utility of artificial ventilation with ENAP. At least part of the benefit of ENAP appears to be secondary to an interaction with the adrenergic nervous system. The pilot survival study, though greatly underpowered, suggests the potential importance of the ENAP treatment in selective clinical conditions associated with cardiovascular collapse.

## Data Availability

The datasets used and/or analyzed during the current study are available from the corresponding author on reasonable request.

## Abbreviations

CPP: Coronary arterial perfusion pressure
ENAP: Expiratory negative airway pressure
ITV: Impedance threshold valve
PAP: Pulmonary arterial systolic pressure
PCWP: Pulmonary capillary wedge pressure
RAP: Right atrial pressure
PVR: Pulmonary vascular resistance
SVR: Systemic vascular resistance
